# 4-Formyl­phenyl 2,3,4,6-tetra-*O*-acetyl-β-d-galactopyran­oside

**DOI:** 10.1107/S1600536811008257

**Published:** 2011-03-09

**Authors:** Rusnah Syahila Duali Hussen, Thorsten Heidelberg, Nasrul Zamani Mohd Rodzi, Seik Weng Ng, Edward R. T. Tiekink

**Affiliations:** a Department of Chemistry, University of Malaya, 50603 Kuala Lumpur, Malaysia

## Abstract

The galactose ring in the title compound, C_21_H_24_O_11_, has a chair conformation with the substituted benzene ring occupying an equatorial position. The crystal packing features C—H⋯O inter­actions that lead to the formation of supra­molecular layers in the *ab* plane.

## Related literature

For the synthesis, see: Benassi *et al.* (2007 ▶); Patil *et al.* (2008 ▶). For the biological activity of related structures, see: Zheng *et al.* (2010 ▶). For the structure of the isomeric allopyran­oside and glucopyran­oside derivatives, see: Ye *et al.* (2009 ▶); Heidelberg *et al.* (2011 ▶). For conformational analysis, see: Cremer & Pople (1975 ▶).
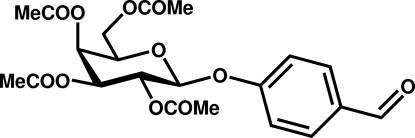


## Experimental

### 

#### Crystal data

C_21_H_24_O_11_
*M* *_r_* = 452.40
Monoclinic, 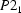

*a* = 11.8358 (4) Å
*b* = 5.6664 (2) Å
*c* = 17.5079 (6) Å
β = 109.616 (4)°
*V* = 1106.05 (7) Å^3^
*Z* = 2
Mo *K*α radiation
μ = 0.11 mm^−1^
*T* = 100 K
0.25 × 0.20 × 0.05 mm

#### Data collection

Agilent Supernova Dual diffractometer with an Atlas detector
Absorption correction: multi-scan (*CrysAlis PRO*; Agilent, 2010 ▶) *T*_min_ = 0.596, *T*_max_ = 1.000
10396 measured reflections
2768 independent reflections
2535 reflections with *I* > 2σ(*I*)
*R*_int_ = 0.051

#### Refinement

*R*[*F* ^2^ > 2σ(*F* ^2^)] = 0.035
*wR*(*F* ^2^) = 0.086
*S* = 1.05
2768 reflections
293 parameters
1 restraint
H-atom parameters constrained
Δρ_max_ = 0.21 e Å^−3^
Δρ_min_ = −0.21 e Å^−3^

### 

Data collection: *CrysAlis PRO* (Agilent, 2010 ▶); cell refinement: *CrysAlis PRO*; data reduction: *CrysAlis PRO*; program(s) used to solve structure: *SHELXS97* (Sheldrick, 2008 ▶); program(s) used to refine structure: *SHELXL97* (Sheldrick, 2008 ▶); molecular graphics: *ORTEP-3* (Farrugia, 1997 ▶) and *DIAMOND* (Brandenburg, 2006 ▶); software used to prepare material for publication: *publCIF* (Westrip, 2010 ▶).

## Supplementary Material

Crystal structure: contains datablocks global, I. DOI: 10.1107/S1600536811008257/ez2236sup1.cif

Structure factors: contains datablocks I. DOI: 10.1107/S1600536811008257/ez2236Isup2.hkl

Additional supplementary materials:  crystallographic information; 3D view; checkCIF report

## Figures and Tables

**Table 1:** Hydrogen-bond geometry (Å, °)

*D*—H⋯*A*	*D*—H	H⋯*A*	*D*⋯*A*	*D*—H⋯*A*
C3—H3⋯O9^i^	1.00	2.39	3.199 (3)	137
C5—H5⋯O9^i^	1.00	2.45	3.268 (3)	139
C10—H10b⋯O3^ii^	0.98	2.46	3.307 (3)	145
C12—H12b⋯O5^iii^	0.98	2.57	3.548 (3)	172
C14—H14c⋯O11^iv^	0.98	2.50	3.415 (4)	155

Symmetry codes: (i) 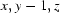; (ii) 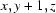; (iii) 
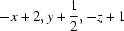
; (iv) 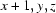.

**Fig. 1.**
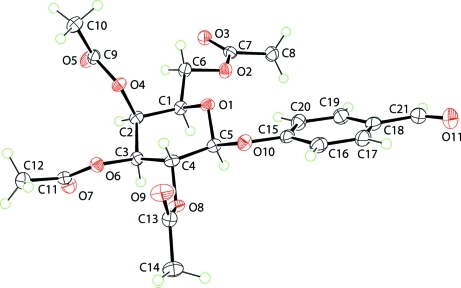
Molecular structure, showing the atom-labelling scheme and displacement ellipsoids at the 50% probability level.

**Fig. 2.**
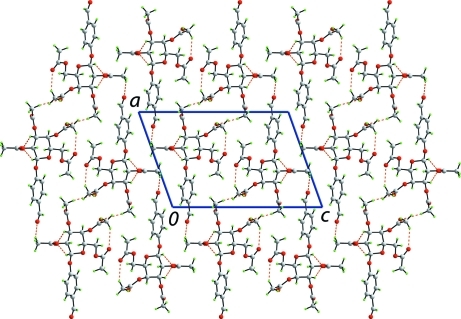
A view in projection down the b axis of the unit-cell contents highlighting the stacking of layers. The C—H···O interactions are shown as orange dashed lines.

## supplementary crystallographic information

### Comment

The title compound, 4-formyl-phenyl 2,3,4,6-tetra-*O*-acetyl-β-D-galactopyranoside, a known species (Benassi *et al.*, 2007; Patil *et al.*, 2008), was prepared as a precursor for the synthesis of galactosylated resveratrol, an anti-oxidizing agent with possible pharmaceutical potential (Zheng *et al.*, 2010).

The structure determination, Fig. 1, confirms the relative stereochemistry. The absolute structure, while not determined experimentally, is based on that of the acetobromogalactose reagent, *i.e. R*, *S*, *S*, *R* and *S* for C1–C5, respectively. The galactose ring has a chair conformation as seen in the puckering parameters (Cremer & Pople, 1975): puckering amplitude (Q) = 0.579 (2) Å, θ = 166.9 (2) °, and φ = 187.6 (10) °. Around the ring, the substituents at the C1, C4 and C5 atoms are equatorial, while at C2 the substituent is axial and that at the C3 atom is biaxial.

The crystal packing is dominated by C–H···O interactions, Table 1, involving all carbonyl atoms, except the O7 atom, as acceptors and either methine- or methyl-H as the donors. These lead to the formation of supramolecular layers that stack along the *c* axis, Fig. 2.

The present report complements the structures reported recently for the isomeric allopyranoside (Ye *et al.*, 2009) and glucopyranoside (Heidelberg *et al.*, 2011) derivatives.

### Experimental

2,3,4,6-Tetra-*O*-acetyl-α-D-galactopyranosyl bromide (2.0 g) and 4-hydroxybenzaldehyde (1.0 g) were dissolved in chloroform (10 ml) and the mixture was treated with an aqueous solution (5 ml) of sodium carbonate (0.9 g) and tetrabutylammonium bromide (0.3 g). The mixture was heated to reflux under vigorous stirring overnight, after which ethyl acetate was added and the organic layer was washed three times with sodium hydroxide solution (1 N) to remove remaining phenols. After drying the solution over magnesium sulfate and evaporation of the solvent, the target product (1.4 g, 60%) was obtained by crystallization from 2-propanol/hexane (2:1).

^1^H-NMR (400 MHz, CDCl_3_): δ 9.93 (s; CHO), 7.85 & 7.11 (AB syst; aromatic 4 H), 5.52 (dd; H2), 5.48 (bd; H4), 5.17 (d; H1), 5.12 (dd; H3), 4.23 (dd; H6a), 4.19–4.10 (m, 2 H; H5, H6b), 2.19–2.02 (3 s, 12 H; Ac); ^3^J_4,5_ = 10.0 Hz, ^3^J_5,6a_ = 5.0 Hz, ^3^J_5,6 b_ = 2.5 Hz, ^2^J_6_ = 12.0 Hz.

### Refinement

Carbon-bound H-atoms were placed in calculated positions (C—H 0.95 to 1.00 Å) and were included in the refinement in the riding model approximation, with *U*_iso_(H) set to 1.2 to 1.5*U*_equiv_(C). In the absence of significant anomalous scattering effects, 1977 Friedel pairs were averaged in the final refinement. The absolute structure was assigned on the basis of that for the acetobromogalactose reagent.

### Crystal data

**Table:**

C_21_H_24_O_11_	*F*(000) = 476
*M**_r_* = 452.40	*D*_x_ = 1.358 Mg m^−^^3^
Monoclinic, *P*2_1_	Mo *K*α radiation, λ = 0.71073 Å
Hall symbol: P 2yb	Cell parameters from 5487 reflections
*a* = 11.8358 (4) Å	θ = 2.5–29.3°
*b* = 5.6664 (2) Å	µ = 0.11 mm^−^^1^
*c* = 17.5079 (6) Å	*T* = 100 K
β = 109.616 (4)°	Prism, colourless
*V* = 1106.05 (7) Å^3^	0.25 × 0.20 × 0.05 mm
*Z* = 2	

### Data collection

**Table:**

Agilent Supernova Dual diffractometer with an Atlas detector	2768 independent reflections
Radiation source: SuperNova (Mo) X-ray Source	2535 reflections with *I* > 2σ(*I*)
Mirror	*R*_int_ = 0.051
Detector resolution: 10.4041 pixels mm^-1^	θ_max_ = 27.5°, θ_min_ = 2.5°
ω scans	*h* = −15→15
Absorption correction: multi-scan (*CrysAlis PRO*; Agilent, 2010)	*k* = −7→6
*T*_min_ = 0.596, *T*_max_ = 1.000	*l* = −22→16
10396 measured reflections	

### Refinement

**Table:**

Refinement on *F*^2^	Primary atom site location: structure-invariant direct methods
Least-squares matrix: full	Secondary atom site location: difference Fourier map
*R*[*F*^2^ > 2σ(*F*^2^)] = 0.035	Hydrogen site location: inferred from neighbouring sites
*wR*(*F*^2^) = 0.086	H-atom parameters constrained
*S* = 1.05	*w* = 1/[σ^2^(*F*_o_^2^) + (0.0424*P*)^2^ + 0.1621*P*] where *P* = (*F*_o_^2^ + 2*F*_c_^2^)/3
2768 reflections	(Δ/σ)_max_ < 0.001
293 parameters	Δρ_max_ = 0.21 e Å^−^^3^
1 restraint	Δρ_min_ = −0.21 e Å^−^^3^

### Special details

**Table:**

Geometry. All e.s.d.'s (except the e.s.d. in the dihedral angle between two l.s. planes) are estimated using the full covariance matrix. The cell e.s.d.'s are taken into account individually in the estimation of e.s.d.'s in distances, angles and torsion angles; correlations between e.s.d.'s in cell parameters are only used when they are defined by crystal symmetry. An approximate (isotropic) treatment of cell e.s.d.'s is used for estimating e.s.d.'s involving l.s. planes.
Refinement. Refinement of *F*^2^ against ALL reflections. The weighted *R*-factor *wR* and goodness of fit *S* are based on *F*^2^, conventional *R*-factors *R* are based on *F*, with *F* set to zero for negative *F*^2^. The threshold expression of *F*^2^ > σ(*F*^2^) is used only for calculating *R*-factors(gt) *etc*. and is not relevant to the choice of reflections for refinement. *R*-factors based on *F*^2^ are statistically about twice as large as those based on *F*, and *R*- factors based on ALL data will be even larger.

### Fractional atomic coordinates and isotropic or equivalent isotropic displacement parameters (Å^2^)

**Table:**

	*x*	*y*	*z*	*U*_iso_*/*U*_eq_	
O1	0.52081 (12)	0.4982 (3)	0.29215 (8)	0.0188 (3)	
O2	0.48738 (13)	0.0896 (3)	0.37091 (9)	0.0240 (4)	
O3	0.55891 (13)	−0.1643 (3)	0.47497 (9)	0.0243 (4)	
O4	0.75837 (12)	0.6298 (3)	0.39447 (8)	0.0184 (3)	
O5	0.86847 (14)	0.3841 (3)	0.49186 (9)	0.0283 (4)	
O6	0.83224 (12)	0.7065 (3)	0.26663 (8)	0.0202 (3)	
O7	0.95682 (14)	0.3988 (3)	0.27801 (9)	0.0271 (4)	
O8	0.61785 (14)	0.7131 (3)	0.12877 (9)	0.0202 (3)	
O9	0.61716 (16)	1.1080 (3)	0.13951 (10)	0.0304 (4)	
O10	0.41232 (13)	0.7100 (3)	0.18039 (8)	0.0213 (3)	
O11	−0.15194 (15)	0.5245 (5)	0.06218 (12)	0.0490 (6)	
C1	0.61385 (17)	0.3269 (4)	0.32349 (12)	0.0180 (4)	
H1	0.6016	0.1990	0.2821	0.022*	
C2	0.73547 (18)	0.4392 (4)	0.33610 (12)	0.0178 (4)	
H2	0.8001	0.3174	0.3549	0.021*	
C3	0.73421 (17)	0.5437 (4)	0.25518 (12)	0.0177 (4)	
H3	0.7421	0.4124	0.2191	0.021*	
C4	0.62145 (17)	0.6840 (4)	0.21135 (11)	0.0177 (4)	
H4	0.6253	0.8420	0.2376	0.021*	
C5	0.50894 (18)	0.5541 (4)	0.21107 (12)	0.0182 (4)	
H5	0.4966	0.4081	0.1772	0.022*	
C6	0.59907 (18)	0.2203 (4)	0.39814 (12)	0.0216 (5)	
H6A	0.6670	0.1138	0.4253	0.026*	
H6B	0.5958	0.3454	0.4368	0.026*	
C7	0.47923 (18)	−0.0968 (4)	0.41586 (12)	0.0205 (4)	
C8	0.35738 (19)	−0.2058 (5)	0.38251 (13)	0.0272 (5)	
H8A	0.3622	−0.3731	0.3974	0.041*	
H8B	0.3277	−0.1909	0.3233	0.041*	
H8C	0.3024	−0.1247	0.4049	0.041*	
C9	0.83088 (18)	0.5785 (4)	0.47134 (12)	0.0195 (4)	
C10	0.8534 (2)	0.7915 (4)	0.52407 (13)	0.0246 (5)	
H10A	0.9152	0.7559	0.5762	0.037*	
H10B	0.7791	0.8375	0.5332	0.037*	
H10C	0.8807	0.9212	0.4976	0.037*	
C11	0.94103 (19)	0.6087 (5)	0.27887 (12)	0.0221 (5)	
C12	1.0356 (2)	0.7935 (5)	0.29539 (14)	0.0301 (6)	
H12A	1.0988	0.7413	0.2746	0.045*	
H12B	1.0701	0.8202	0.3540	0.045*	
H12C	1.0003	0.9407	0.2684	0.045*	
C13	0.61949 (19)	0.9346 (4)	0.10083 (13)	0.0208 (5)	
C14	0.6223 (2)	0.9316 (5)	0.01601 (13)	0.0284 (5)	
H14A	0.6497	1.0852	0.0034	0.043*	
H14B	0.5416	0.8993	−0.0219	0.043*	
H14C	0.6774	0.8081	0.0111	0.043*	
C15	0.29775 (18)	0.6182 (5)	0.16131 (12)	0.0214 (5)	
C16	0.2083 (2)	0.7649 (5)	0.11247 (13)	0.0269 (5)	
H16	0.2281	0.9110	0.0935	0.032*	
C17	0.0891 (2)	0.6936 (5)	0.09193 (14)	0.0310 (6)	
H17	0.0271	0.7924	0.0588	0.037*	
C18	0.0601 (2)	0.4804 (5)	0.11914 (13)	0.0282 (5)	
C19	0.1514 (2)	0.3361 (5)	0.16730 (13)	0.0275 (5)	
H19	0.1317	0.1889	0.1856	0.033*	
C20	0.27087 (19)	0.4037 (4)	0.18905 (13)	0.0246 (5)	
H20	0.3328	0.3050	0.2222	0.030*	
C21	−0.0667 (2)	0.4052 (6)	0.09904 (14)	0.0369 (6)	
H21	−0.0816	0.2530	0.1163	0.044*	

### Atomic displacement parameters (Å^2^)

**Table:**

	*U*^11^	*U*^22^	*U*^33^	*U*^12^	*U*^13^	*U*^23^
O1	0.0204 (7)	0.0207 (8)	0.0147 (7)	0.0044 (6)	0.0053 (6)	0.0017 (6)
O2	0.0208 (7)	0.0267 (9)	0.0219 (7)	−0.0005 (7)	0.0037 (6)	0.0064 (7)
O3	0.0241 (7)	0.0252 (9)	0.0233 (7)	−0.0009 (7)	0.0075 (6)	0.0057 (7)
O4	0.0216 (7)	0.0151 (7)	0.0159 (6)	0.0016 (6)	0.0025 (6)	−0.0009 (6)
O5	0.0353 (9)	0.0198 (9)	0.0226 (7)	0.0033 (7)	0.0000 (7)	0.0025 (7)
O6	0.0205 (7)	0.0175 (8)	0.0231 (7)	−0.0019 (6)	0.0081 (6)	−0.0008 (6)
O7	0.0248 (8)	0.0258 (10)	0.0309 (8)	0.0023 (7)	0.0095 (7)	0.0016 (8)
O8	0.0305 (8)	0.0153 (8)	0.0149 (7)	0.0001 (7)	0.0078 (6)	−0.0004 (6)
O9	0.0498 (10)	0.0161 (8)	0.0268 (8)	−0.0001 (8)	0.0148 (8)	0.0009 (8)
O10	0.0211 (7)	0.0181 (8)	0.0220 (7)	0.0049 (6)	0.0035 (6)	0.0020 (7)
O11	0.0240 (9)	0.0789 (17)	0.0400 (11)	0.0101 (10)	0.0052 (8)	−0.0055 (11)
C1	0.0201 (9)	0.0133 (10)	0.0193 (9)	0.0013 (9)	0.0048 (8)	0.0014 (8)
C2	0.0214 (10)	0.0131 (11)	0.0181 (9)	0.0008 (8)	0.0054 (8)	−0.0015 (8)
C3	0.0189 (10)	0.0142 (11)	0.0200 (10)	−0.0020 (8)	0.0064 (8)	−0.0014 (8)
C4	0.0238 (10)	0.0151 (11)	0.0144 (9)	0.0020 (9)	0.0065 (8)	0.0005 (8)
C5	0.0218 (10)	0.0146 (11)	0.0168 (9)	0.0034 (8)	0.0048 (8)	0.0008 (8)
C6	0.0197 (10)	0.0224 (12)	0.0204 (10)	−0.0029 (9)	0.0039 (8)	0.0039 (9)
C7	0.0219 (10)	0.0206 (11)	0.0217 (10)	−0.0018 (9)	0.0107 (9)	−0.0013 (9)
C8	0.0241 (11)	0.0309 (14)	0.0266 (11)	−0.0053 (10)	0.0087 (9)	0.0010 (11)
C9	0.0172 (9)	0.0210 (12)	0.0180 (9)	−0.0019 (9)	0.0027 (8)	0.0022 (9)
C10	0.0272 (11)	0.0224 (12)	0.0200 (10)	0.0011 (10)	0.0024 (9)	−0.0004 (10)
C11	0.0230 (10)	0.0270 (13)	0.0167 (9)	−0.0005 (10)	0.0072 (8)	−0.0005 (10)
C12	0.0255 (11)	0.0358 (15)	0.0290 (11)	−0.0071 (11)	0.0092 (10)	−0.0055 (11)
C13	0.0214 (10)	0.0174 (12)	0.0222 (10)	0.0005 (9)	0.0056 (9)	0.0034 (9)
C14	0.0380 (12)	0.0267 (14)	0.0215 (10)	0.0039 (11)	0.0113 (10)	0.0041 (10)
C15	0.0213 (10)	0.0248 (12)	0.0170 (9)	0.0027 (9)	0.0050 (8)	−0.0051 (9)
C16	0.0297 (12)	0.0280 (13)	0.0216 (10)	0.0069 (10)	0.0067 (10)	0.0003 (10)
C17	0.0243 (11)	0.0433 (16)	0.0222 (10)	0.0113 (11)	0.0036 (9)	−0.0008 (11)
C18	0.0252 (11)	0.0389 (15)	0.0201 (10)	0.0029 (11)	0.0070 (9)	−0.0085 (11)
C19	0.0269 (11)	0.0276 (13)	0.0279 (11)	−0.0027 (10)	0.0091 (10)	−0.0070 (10)
C20	0.0227 (10)	0.0235 (12)	0.0250 (10)	0.0036 (10)	0.0046 (9)	−0.0003 (10)
C21	0.0257 (12)	0.0567 (19)	0.0284 (12)	0.0004 (13)	0.0092 (10)	−0.0121 (13)

### Geometric parameters (Å, °)

**Table:**

O1—C5	1.415 (2)	C7—C8	1.496 (3)
O1—C1	1.432 (2)	C8—H8A	0.9800
O2—C7	1.340 (3)	C8—H8B	0.9800
O2—C6	1.449 (3)	C8—H8C	0.9800
O3—C7	1.205 (3)	C9—C10	1.488 (3)
O4—C9	1.362 (2)	C10—H10A	0.9800
O4—C2	1.448 (2)	C10—H10B	0.9800
O5—C9	1.197 (3)	C10—H10C	0.9800
O6—C11	1.351 (3)	C11—C12	1.489 (3)
O6—C3	1.443 (2)	C12—H12A	0.9800
O7—C11	1.205 (3)	C12—H12B	0.9800
O8—C13	1.350 (3)	C12—H12C	0.9800
O8—C4	1.441 (2)	C13—C14	1.497 (3)
O9—C13	1.199 (3)	C14—H14A	0.9800
O10—C15	1.384 (3)	C14—H14B	0.9800
O10—C5	1.401 (2)	C14—H14C	0.9800
O11—C21	1.206 (3)	C15—C20	1.385 (3)
C1—C6	1.502 (3)	C15—C16	1.391 (3)
C1—C2	1.521 (3)	C16—C17	1.393 (3)
C1—H1	1.0000	C16—H16	0.9500
C2—C3	1.531 (3)	C17—C18	1.384 (4)
C2—H2	1.0000	C17—H17	0.9500
C3—C4	1.521 (3)	C18—C19	1.391 (3)
C3—H3	1.0000	C18—C21	1.484 (3)
C4—C5	1.520 (3)	C19—C20	1.389 (3)
C4—H4	1.0000	C19—H19	0.9500
C5—H5	1.0000	C20—H20	0.9500
C6—H6A	0.9900	C21—H21	0.9500
C6—H6B	0.9900		
			
C5—O1—C1	109.93 (14)	H8B—C8—H8C	109.5
C7—O2—C6	116.57 (16)	O5—C9—O4	122.7 (2)
C9—O4—C2	116.39 (17)	O5—C9—C10	126.07 (19)
C11—O6—C3	116.03 (17)	O4—C9—C10	111.22 (19)
C13—O8—C4	118.07 (17)	C9—C10—H10A	109.5
C15—O10—C5	117.57 (17)	C9—C10—H10B	109.5
O1—C1—C6	107.86 (16)	H10A—C10—H10B	109.5
O1—C1—C2	109.91 (17)	C9—C10—H10C	109.5
C6—C1—C2	115.02 (17)	H10A—C10—H10C	109.5
O1—C1—H1	107.9	H10B—C10—H10C	109.5
C6—C1—H1	107.9	O7—C11—O6	123.1 (2)
C2—C1—H1	107.9	O7—C11—C12	125.9 (2)
O4—C2—C1	111.00 (16)	O6—C11—C12	110.9 (2)
O4—C2—C3	107.82 (17)	C11—C12—H12A	109.5
C1—C2—C3	108.23 (16)	C11—C12—H12B	109.5
O4—C2—H2	109.9	H12A—C12—H12B	109.5
C1—C2—H2	109.9	C11—C12—H12C	109.5
C3—C2—H2	109.9	H12A—C12—H12C	109.5
O6—C3—C4	105.32 (16)	H12B—C12—H12C	109.5
O6—C3—C2	111.18 (16)	O9—C13—O8	123.49 (19)
C4—C3—C2	113.79 (16)	O9—C13—C14	125.6 (2)
O6—C3—H3	108.8	O8—C13—C14	110.91 (19)
C4—C3—H3	108.8	C13—C14—H14A	109.5
C2—C3—H3	108.8	C13—C14—H14B	109.5
O8—C4—C5	108.73 (16)	H14A—C14—H14B	109.5
O8—C4—C3	106.93 (15)	C13—C14—H14C	109.5
C5—C4—C3	111.57 (17)	H14A—C14—H14C	109.5
O8—C4—H4	109.9	H14B—C14—H14C	109.5
C5—C4—H4	109.9	O10—C15—C20	124.53 (19)
C3—C4—H4	109.9	O10—C15—C16	113.9 (2)
O10—C5—O1	108.61 (15)	C20—C15—C16	121.5 (2)
O10—C5—C4	107.29 (17)	C15—C16—C17	118.7 (2)
O1—C5—C4	108.26 (16)	C15—C16—H16	120.6
O10—C5—H5	110.9	C17—C16—H16	120.6
O1—C5—H5	110.9	C18—C17—C16	120.8 (2)
C4—C5—H5	110.9	C18—C17—H17	119.6
O2—C6—C1	106.20 (16)	C16—C17—H17	119.6
O2—C6—H6A	110.5	C17—C18—C19	119.3 (2)
C1—C6—H6A	110.5	C17—C18—C21	121.1 (2)
O2—C6—H6B	110.5	C19—C18—C21	119.5 (3)
C1—C6—H6B	110.5	C20—C19—C18	121.1 (2)
H6A—C6—H6B	108.7	C20—C19—H19	119.5
O3—C7—O2	124.39 (19)	C18—C19—H19	119.5
O3—C7—C8	125.4 (2)	C15—C20—C19	118.6 (2)
O2—C7—C8	110.25 (18)	C15—C20—H20	120.7
C7—C8—H8A	109.5	C19—C20—H20	120.7
C7—C8—H8B	109.5	O11—C21—C18	124.3 (3)
H8A—C8—H8B	109.5	O11—C21—H21	117.8
C7—C8—H8C	109.5	C18—C21—H21	117.8
H8A—C8—H8C	109.5		
			
C5—O1—C1—C6	−163.31 (17)	C3—C4—C5—O1	54.5 (2)
C5—O1—C1—C2	70.6 (2)	C7—O2—C6—C1	151.59 (18)
C9—O4—C2—C1	100.1 (2)	O1—C1—C6—O2	66.5 (2)
C9—O4—C2—C3	−141.53 (17)	C2—C1—C6—O2	−170.44 (17)
O1—C1—C2—O4	61.5 (2)	C6—O2—C7—O3	−2.2 (3)
C6—C1—C2—O4	−60.5 (2)	C6—O2—C7—C8	177.27 (18)
O1—C1—C2—C3	−56.7 (2)	C2—O4—C9—O5	−4.5 (3)
C6—C1—C2—C3	−178.60 (18)	C2—O4—C9—C10	176.56 (17)
C11—O6—C3—C4	−158.49 (16)	C3—O6—C11—O7	2.3 (3)
C11—O6—C3—C2	77.8 (2)	C3—O6—C11—C12	−176.14 (16)
O4—C2—C3—O6	44.2 (2)	C4—O8—C13—O9	−4.2 (3)
C1—C2—C3—O6	164.33 (16)	C4—O8—C13—C14	176.78 (17)
O4—C2—C3—C4	−74.5 (2)	C5—O10—C15—C20	17.7 (3)
C1—C2—C3—C4	45.6 (2)	C5—O10—C15—C16	−164.09 (18)
C13—O8—C4—C5	120.0 (2)	O10—C15—C16—C17	−177.76 (19)
C13—O8—C4—C3	−119.44 (19)	C20—C15—C16—C17	0.5 (3)
O6—C3—C4—O8	73.70 (19)	C15—C16—C17—C18	−0.3 (3)
C2—C3—C4—O8	−164.29 (17)	C16—C17—C18—C19	−0.2 (3)
O6—C3—C4—C5	−167.54 (16)	C16—C17—C18—C21	178.7 (2)
C2—C3—C4—C5	−45.5 (2)	C17—C18—C19—C20	0.6 (3)
C15—O10—C5—O1	−74.1 (2)	C21—C18—C19—C20	−178.3 (2)
C15—O10—C5—C4	169.11 (16)	O10—C15—C20—C19	177.9 (2)
C1—O1—C5—O10	176.18 (16)	C16—C15—C20—C19	−0.2 (3)
C1—O1—C5—C4	−67.6 (2)	C18—C19—C20—C15	−0.4 (3)
O8—C4—C5—O10	−70.80 (19)	C17—C18—C21—O11	−3.8 (4)
C3—C4—C5—O10	171.51 (15)	C19—C18—C21—O11	175.1 (2)
O8—C4—C5—O1	172.16 (16)		

### Hydrogen-bond geometry (Å, °)

**Table:**

*D*—H···*A*	*D*—H	H···*A*	*D*···*A*	*D*—H···*A*
C3—H3···O9^i^	1.00	2.39	3.199 (3)	137
C5—H5···O9^i^	1.00	2.45	3.268 (3)	139
C10—H10b···O3^ii^	0.98	2.46	3.307 (3)	145
C12—H12b···O5^iii^	0.98	2.57	3.548 (3)	172
C14—H14c···O11^iv^	0.98	2.50	3.415 (4)	155

Symmetry codes: (i) *x*, *y*−1, *z*; (ii) *x*, *y*+1, *z*; (iii) −*x*+2, *y*+1/2, −*z*+1; (iv) *x*+1, *y*, *z*.
